# Considerations for Sample Preparation Using Size-Exclusion Chromatography for Home and Synchrotron Sources

**DOI:** 10.1007/978-981-10-6038-0_3

**Published:** 2017-12-08

**Authors:** Robert P. Rambo

**Affiliations:** Diamond Light Source Ltd., Harwell Science & Innovation Campus, Didcot, OX11 0DE UK

**Keywords:** SEC, MALS, QELS, Total scattered intensity, Additives, Stability, Aggregation, Monodispersity, Sucrose, Phosphate

## Abstract

The success of a SAXS experiment for structural investigations depends on two precise measurements, the sample and the buffer background. Buffer matching between the sample and background can be achieved using dialysis methods but in biological SAXS of monodisperse systems, sample preparation is routinely being performed with size exclusion chromatography (SEC). SEC is the most reliable method for SAXS sample preparation as the method not only purifies the sample for SAXS but also almost guarantees ideal buffer matching. Here, I will highlight the use of SEC for SAXS sample preparation and demonstrate using example proteins that SEC purification does not always provide for ideal samples. Scrutiny of the SEC elution peak using quasi-elastic and multi-angle light scattering techniques can reveal hidden features (heterogeneity) of the sample that should be considered during SAXS data analysis. In some cases, sample heterogeneity can be controlled using a small molecule additive and I outline a simple additive screening method for sample preparation.

## Introduction

Structural investigations of biological systems in the solution-state are investigations made from an ensemble of macromolecular particles. In biological, solution-state SAXS, the ensemble is composed of thousands of billions of macromolecules in various interchangeable, conformational states (Rambo and Tainer 2010a, b). Since domain motions range from micro- to milli-seconds (Henzler-Wildman et al. 2007), a solution-state SAXS measurement is often an observation of the thermodynamic state due to the X-ray exposure times being much greater than the internal motions of the particle. The distribution of macromolecules across this conformational landscape is determined by the buffer composition and temperature that defines the sample environment.

The SAXS signal is a direct observation of this conformational landscape. If the landscape is broad and diverse, interpreting the SAXS signal using a single atomistic model will be difficult and likewise, the information quality of any *ab initio* model will be low. This type of conformational heterogeneity is difficult to assess but can be influenced by changing the composition of the buffer (Rambo and Tainer 2010a, b). Similarly, mass heterogeneity due to multimerization, aggregation or low purity will reduce the information quality of the SAXS signal and confound the structural interpretation (Jacques and Trewhella 2010). These issues highlight a fundamental property of solution-state SAXS and that is *everything scatters* in the sample. Unlike NMR and X-ray crystallography where heterogeneity will broaden and weaken the desired structural signal, heterogeneity contributes directly to the SAXS signal whose contribution is proportional to mass and concentration. Therefore, any structural modelling using SAXS data must be made from data collected from samples that are well-characterized and optimized for monodispersity and homogeneity (Rambo and Tainer 2013).

**Fig. 3.1:**
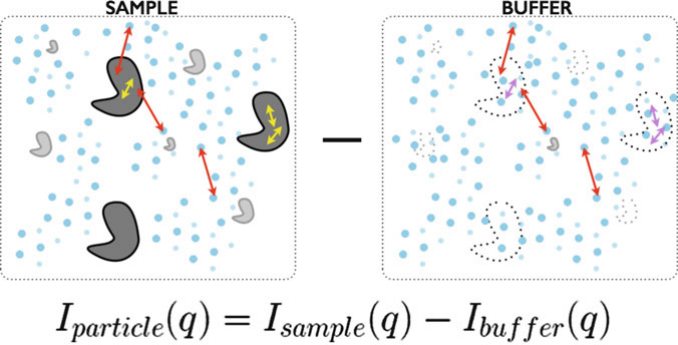
SAXS as a difference measurement. SAXS requires two precise measurements *1* sample (particles + buffer) and *2* buffer background. For the sample, the scattering is the result of the dissolved particles, solute that participates in the hydration of the particle, and bulk solvent. For the buffer, the scattering will be the result of the bulk solvent, solute that will participate in the particle hydration, and the excluded volume. The excluded volume is the imprint of the particle in the buffer whose electron density is bulk solvent. Under dilute, monodispersed particle conditions, the observed SAXS intensity will be approximated as the scattering from a single particle scaled by concentration with corrections due to the excluded volume and hydration. At low resolution, the d-spacing vectors (2π/q) (*red double arrows*) are large and can only exists across the particle-solvent boundary whereas at higher resolutions, the d-spacing vector is smaller (*yellow and magenta double arrows*) and can exists within a single particle. To accurately minimize contributions from the bulk solvent, the composition of the bulk solvent in both the sample and background must be identical

Unfortunately, the quality of the measured SAXS signal is not fully determined by sample heterogeneity. The actual SAXS signal of the ensemble is taken as the difference (Fig. 3.1) between the measured SAXS curve of the sample (i.e., particle and buffer) and the background (buffer only). Matching the buffer between the sample and background is critical to the accuracy of the recovered SAXS curve. Particularly at high scattering vectors (q), poor buffer matching often leads to under- or over-subtraction and errors in subtraction will limit the resolution of the SAXS experiment. If not properly identified and removed from the recovered SAXS curve during post-processing, the systematic contributions from the mismatch can increase the false discovery rates in modelling and introduce artefacts in the P(r)-distribution.

An efficient and readily available technique that can assess sample quality and provide a reliable method for buffer matching is size exclusion chromatography (SEC). SEC chromatographic separation is based principally on the ability of the macromolecules to move through the pores of the chromatographic resin. If the macromolecule is physically larger than the pores, it will be excluded by the resin and elute relatively early from the SEC column whereas a macromolecule that is smaller than the pores will reside within the column longer and elute later. The standard method for monitoring an SEC chromatographic separation is UV absorption which exploits the absorption properties of the peptide backbone and aromatic rings of common amino and nucleic acids. Absorption based methods only inform on particle concentration. Regardless of size, an elution profile should be nearly symmetric for a sample consisting of homogenous and monodisperse particles. Any peak asymmetry should not go unnoticed and can be indicative of particle-column interaction, multimerization or heterogeneity. Furthermore, the use of native gels to assert a sample is free of aggregation should be avoided as the method of detection is unreliable for obvious reasons.

**Fig. 3.2:**
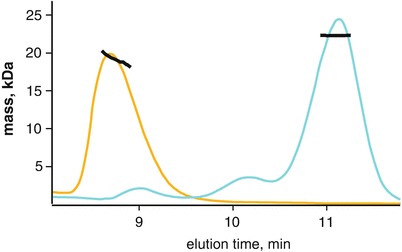
Anomalously eluting proteins by size exclusion chromatography. The Stoke’s radius is a spherical approximation of a particle that scales linearly with globular proteins. Using proteins of known mass, an SEC column can be calibrated where elution volume (time) correlates with protein mass. This method makes a critical assumption regarding the globularity of the particle and is often erroneous with asymmetric particles. Here, the globular particle xylanase (21 kDa) elutes (*cyan*) with a stable MALS mass (y-axis, kDa) across the main peak. In comparison, is a novel protein that has a smaller MALS mass than xylanase but elutes earlier. The protein was determined to be highly asymmetric with high mass heterogeneity. In the absence of MALS, the peak mass would have been over-estimated

SEC is suitable for a wide range of macromolecular masses (10–10,000 kDa) and shapes. For globular proteins, there is a linear relationship between mass and the physical dimensions of the particle. Using a set of standards, i.e., proteins with known mass and dimensions, an SEC column can be calibrated such that the elution time corresponds to the mass of the particle. This technique assumes the unknown particle can be approximated by a simple sphere whose radius (Stoke’s radius) scales linearly with mass. For asymmetric or elongated particles, a calibrated SEC column will give erroneous mass estimates, since it is essentially the largest dimension of the particle that determines how the particle will travel through the column (Fig. 3.2).

Resolving this ambiguity between mass and particle dimensions can only be made using a scattering technique such as multi-angle light scattering (MALS) or SAXS. Here, scattering measurements are made at time points along the SEC run either by fractionation of the elution or by coupling the MALS (SEC-MALS) or SAXS (SEC-SAXS) instrument directly inline with the SEC (Perez and Nishino 2012; Gillis et al. 2014; Jeffries et al. 2016; Meisburger et al. 2016). Direct coupling of the SEC to the scattering instrument has proven to be optimal for accurate scattering measurements of the background and sample. In MALS, it is the intensity of the scattered light by the particle that is used to determine molecular weight. This intensity must be properly normalized by the particle’s concentration whereas in SAXS, it is the angular dependence on the scattered intensity that is used to describe the shape and mass of the particle.

## Refractive Index, Ultra-Violet and Light Scattering

**Fig. 3.3:**
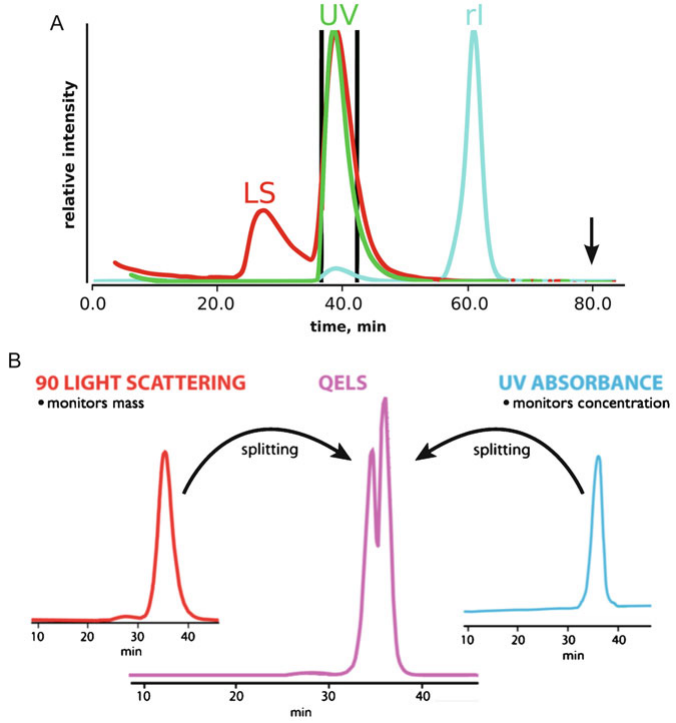
Light scattering, UV and refractive index detection. (**a**) Comparing all three signals can reveal hidden features of a sample. Light scattering (LS) intensity is directly proportional to the squared mass of a particle. The presence of a LS signal in the void (25 min) suggests the presence of large aggregates. In the SEC separation of dissolved glucose isomerase crystals, the samples contained significant amounts of aggregation that were not detectable by UV (flat *green* curve at 25 min). The dissolved crystals contained high concentrations of ammonium sulfate leading to a strong peak in the refractive index (rI) detector near the end of the column run (60 min) as the small molecules eluted off the column. It is recommended that buffers collected for SAXS be obtained at 1.5 column volumes (*black arrow*) where the eluent has stabilized. (**b**) Neither LS nor UV will be sensitive to particle conformation, subtle differences in a sample can be glimpsed by examining time-resolved LS measurements (QELS) across the elution peak. Here, QELS will be proportional to particle conformation and for the PYR1 protein, initial analysis of the protein always revealed a split QELS peak. After 2 weeks, the QELS peaks would resolve to a single peak and it was surmised the splitting was due to an isomerization of a proline residue

Standard UV absorption (A_260 nm, 280 nm_) detectors found on most SEC instruments are not sufficient to monitor all classes of biomolecules or biomolecules with exceedingly low extinction coefficients. Alternatively, a refractive index (rI) detector can be used to reliably monitor particle concentration in a wide range of buffer conditions. The rI detector measures the bending of light between a reference cell (buffer only) and flow cell (SEC eluent). The differential refractive index detector will register a signal as the composition of the flow cell changes relative to the reference cell (Fig. 3.3). The detector can demonstrate small variations in refractive index due to concentration differences in dissolved gases, salts and particle concentration. For proteins and nucleic acids, the refractive index is nearly constant, irrespective of the primary, secondary or tertiary structure of the biopolymer. In contrast, UV absorption detection requires the appropriate chromophore to be present in the biopolymer whose extinction coefficient will vary with the hydrophobic environment of the chromophore. Accurate particle concentration is critical to SEC-MALS and refractive index detectors are the preferred method for concentration determination (Wyatt 1993; Tarazona and Saiz 2003).

SEC-MALS is the most reliable method for assessing mass homogeneity of an SEC elution peak. The MALS instrument contains several detectors arranged in a circle around a flow cell that measures the intensity of scattered light from a laser source. In some instruments, at least one of the detectors can perform time-resolved measurements allowing for quasi-elastic light scattering (QELS) observations. QELS monitors how the solution sparkles with time and it is the decay rate of the sparkling that is proportional to particle dimension of radius-of-hydration (r_H_). QELS will be sensitive to particle conformation and QELS measurements across an elution peak can provide additional information on conformational heterogeneity (Minton 2016).

## Hidden Features (Known Unknowns)

The SEC elution profile of a sample can provide information regarding mass and conformational heterogeneity. Both mass and conformational heterogeneity, as well as particle-resin interaction, will cause an asymmetry in the SEC elution peak. However, the ability to discern these types of heterogeneities rely on how the chromatographic separation is being monitored (Brookes et al. 2013; Meisburger et al. 2016).

Since UV absorption (A_280_) detectors mainly inform on particle concentration and light scattering based methods inform on mass, shape and concentration, an integrated approach that combines both types of measurements can provide a thorough characterization of the sample (Fig. 3.3a). The SEC elution begins with the column’s void volume. The void volume is the volume of elution since sample injection that contains particles too large to be partitioned by the SEC resin. Large aggregates in a sample may go un-detected by UV methods as the concentrations may be too low for detection. However, scattering intensity is proportional to the squared mass suggesting MALS or SAXS will be most sensitive to the presence of large aggregates.

SEC-MALS analysis of dissolved glucose isomerase (GI) crystals (Fig. 3.3a) shows these contrasting features between UV and light scattering. The injected sample demonstrates a large scattering peak at the void volume with essentially no UV signal. The lack of a notable UV signal near the void volume would give the false confidence that the sample was free of aggregation. Therefore, it is recommended that in the absence of an additional scattering detector, any observed deflection of the UV signal near the void volume should be considered significant. In these cases, if the sample is being prepared for SAXS, extensive centrifugation or the use of a spin-filter may be necessary to remove the aggregation prior to data collection (Hura et al. 2009).

Furthermore, the SEC-MALS analysis of GI utilized a refractive index detector. As discussed previously, the refractive index detector is sensitive to differences in the chemical composition of the running buffer as measured against the reference cell. Due to small differences in salt, glycerol or dissolved gases, it can be expected that the buffer composition of the injected sample will not be identical to the SEC running buffer thereby causing a notable signal in the refractive index detector near the end of the column run. Since these small molecules are invisible to UV absorption, the UV signal near the end of the column volume would appear flat giving the false impression that a background sample could be taken. While it is recommended that samples prepared for SAXS by SEC use the same running buffer as background, it is critical to the accuracy of the background subtraction that the buffer collected for the background measurement occur at least 1.5 column volumes after injection (Fig. 3.3a).

In the analysis of an SEC elution peak, the shape of the peak profile is the most informative method for indicating possible sample heterogeneity. Elution peaks that are asymmetric can suggest mass heterogeneity or particle-column interactions. However, conformational heterogeneity that is stable to partitioning can be more difficult to assess unless the structural differences are large enough to produce significant differences in the Stoke’s radius of the different conformations. Here, monitoring the elution peak using QELS or by SAXS can provide additional information to determine the cause of the asymmetry in the elution peak (Fig. 3.3b). SEC-MALS with QELS studies performed on PYR1 (Nishimura et al. 2009), a 42 kDa protein, demonstrated the slightest asymmetry in the MALS and UV absorbance peaks. The MALS mass was consistent across the elution peak suggesting a homogenous sample; however, the QELS measurements showed a splitting of the peak (Fig. 3.3b) suggesting two distinct conformations were present in the single elution peak. SAXS data collected on the peak could not be fully explained by the crystal structure unless the model fitting was performed on the lagging side of the elution peak. In the absence of QELS or MALS information, a comparison of the individual SAXS frames from the leading and lagging sides of the elution profile must be inspected. At the very least, conformational heterogeneity would show the leading side to be larger than the lagging side in terms of R_g_ and possibly d_max_. Due to the thorough characterization of the sample, a multi-model fit would be necessitated to fully explain the SAXS curve.

**Fig. 3.4:**
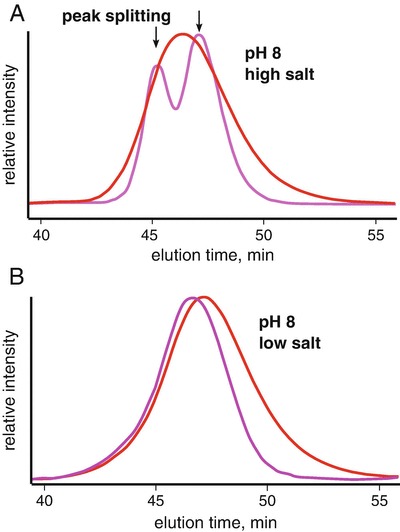
Conformational changes by QELS. Protein conformations are often controlled by the environment. At low pH, protonation of histidines residues change the charge distribution of the protein and can stabilize an alternate conformation. Similarly, salt can provide significant charge shielding to induce the same effects. (**a**) At pH 8 and high salt, it was observed that MALS (*red*) and QELS (*magenta*) analysis of glucose isomerase (GI) leads to a splitting of the QELS peak (*magenta*). (**b**) Resolution of split peak at lower ionic strengths. Close inspection of the MALS peak in high salt shows a slight asymmetry in the elution peak. Note that the QELS and LS signals are shifted for clarity

A similar peak splitting was observed for GI (Fig. 3.4). GI was commonly used in the Tainer laboratory (Classen et al. 2013) as a mass standard for MALS calibrations. It was noticed that in moderately high pH and salt conditions, the QELS data would demonstrate a split peak. The peak splitting would disappear by lowering the salt concentration suggesting the conformational states of the protein could readily be influenced by adjusting the composition of the buffer. Similar observations were made for BSA where at pH >7.5 in PBS buffer, BSA would show a severe, asymmetric elution profile. Lowering the pH or by adding 1% sucrose to the buffer would stabilize the protein to partitioning producing the canonical monomer-dimer SEC profile of BSA.

## Influence

**Fig. 3.5:**
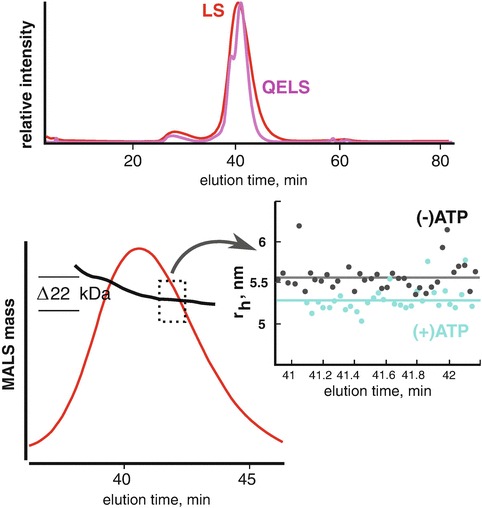
Stable mass heterogeneity. SEC-MALS/QELS analysis of a 185 kDa ATP binding protein reveal a leading shoulder in the QELS (*magenta*). The shoulder corresponded to a larger mass species by MALS (*black line*,*lower left panel*). To test if the protein was responsive to ATP, SEC-MALS/QELS was performed in the presence of 500 uM ATP-vanadate. The (+)ATP state showed a measurable and consistent decrease in radius-of-hydration (r_H_) by QELS (*cyan*) indicating the protein undergoes as a significant conformational change. A small change in r_H_ would translate into an observable change by SAXS

The GI and PYR1 asymmetric elution peaks were due to conformational heterogeneity that could be influenced by the composition of the buffer. This type of conformational heterogeneity was stable to partitioning and produced the peak splitting in the QELS data. Nonetheless, heterogeneity can involve both conformation and mass. Mass heterogeneity is readily detected by MALS and will cause a negative slope in the mass distribution across the elution peak. The mass heterogeneity may be due to the particle in rapid equilibrium with higher order states or due to small truncations of component domains. If the heterogeneity is stable to partitioning, then the MALS data would demonstrate distinct steps in the mass distribution with distinct peaks in the QELS profile (Fig. 3.5). SEC-MALS/QELS studies on a 185 kDa ATP motor protein showed such a profile. The QELS profile contained a leading, shoulder peak suggesting the elution peak contained at least two structurally distinct species. Further analysis of the MALS information showed the shoulder peak was ~22 kDa larger than the lagging side of the elution peak. We speculated the mass difference was due to limited proteolysis of the protein during purification. To test if the protein was responsive to ATP, the SEC-MALS/QELS was repeated with the protein incubated in ATP-vanadate. The vanadate locks down the protein in a phosphoryl-transfer transition state (Davies and Hol 2004) and for a motor protein, binding should demonstrate a notable conformational change. QELS results showed a decrease in the radius-of-hydration upon incubation suggesting the protein was competent to ATP binding and hydrolysis. While the sample is a mixture and remains unresolved during the SEC separation, SAXS data collected on the protein in the bound and unbound states would still be informative. In a SEC-SAXS experiment, a comparative SAXS analysis from the lagging side that uses the P(r)-distributions would characterize the conformational change in terms of compactness and dimensions. It can be expected that a measured decrease in r_H_ would produce a notable decrease in R_g_.

The 185 kDa protein demonstrated a compound heterogeneity involving both mass and conformation. The larger mass species was stable to partitioning by SEC thereby producing a distinct step in the MALS mass distribution across the elution peak. However, heterogeneity could be due to a rapid equilibrium between states such that the partitioning is characterized by a broad and asymmetric elution peak. MALS and QELS analysis will show a slope across the elution peak and likewise, SEC-SAXS would demonstrate a disagreement between the leading and lagging sides of the elution peak. This type of heterogeneity is particularly nefarious and suggests the biological particles are not stable to partitioning down the SEC column.

In macromolecular crystallography, conditions are sought to promote particle-particle interactions and often, the macromolecules are purified to high concentrations in a minimal buffer. These conditions may not be suitable for SAXS and can be the cause of the compound heterogeneity described above. Solution-state structural studies require buffer conditions that are stabilizing to the particle. For nucleic acid binding proteins, we have found phosphate and sucrose to be excellent additives that stabilize the protein while minimizing column-particle interactions. In most cases, nucleic acid binding proteins interact with nucleic acids through the sugar-phosphate backbone and in the absence of nucleic acids, these proteins may be charged imbalanced through the residues arginine and lysine. In the apo-state, the addition of 1% sucrose and phosphate can make a poor SAXS sample into an excellent, well-behaved SAXS sample.

**Fig. 3.6:**
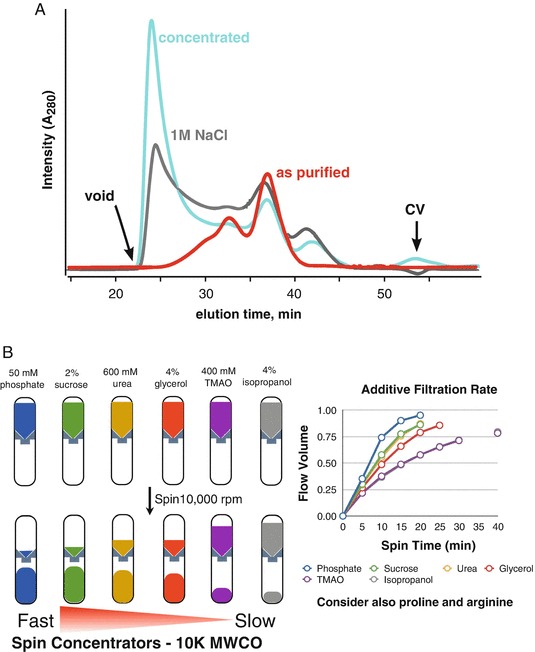
Sample instability during concentrating. Protein aggregation during concentrating is a routinely encountered problem and leads to exceedingly long concentrating times when using a spin-concentrator. The issue may not be relevant for most biochemistry experiments, however, it is a critical problem for SAXS. The WRN exonuclease domain was purified and concentrated in 20 mM Tris (7.6), 200 mM NaCl, and 5 mM beta-mercaptoethanol. (**a**) The protein was subjected to SEC analysis using a Superdex 200 PC 3.2 column in the same buffer (*red trace*). Upon concentrating the protein, the injected sample produced a significant UV signal in the void volume (*gray trace*). Concentrating the protein in buffer with 1 M NaCl increased the aggregation peak (*cyan trace*). (**b**) Additive screen using 10 K MWCO spin-concentrators. Various additives were added to the buffer and used to dilute the protein. For each additive, the filtration rate (volume of material that flowed through during centrifugation) was recorded at 5 min intervals. Samples contained phosphate (*blue*) and sucrose (*green*) had the fastest flow rates (Figure adapted from Kevin Dyer, Advanced Light Source, SIBYLS beamline, Berkeley, CA)

The effects of additives must be evaluated using a suitable assay (Han et al. 2007; Leibly et al. 2012). If using SEC, the additives can be added to the running buffer, but this method will take hours per additive as the SEC column will have to be equilibrated for each additive. Another method for screening the effects of additives can be performed using micro-spin concentrators. The Tainer group had successfully solved the crystal structure of the exonuclease domain from the DNA repair protein WRN (Perry et al. 2006). The functional state of the domain in solution was unknown with some results suggesting the protein was a trimer (Choi et al. 2007). The domain, as purified, would aggregate during spin-concentrating causing the flow-rate to be exceptionally slow with a significant loss of material. Concentrating the protein in high salt (1 M NaCl) failed to stabilize the protein leading to a large redistribution of the protein into the void volume (Fig. 3.6a). Therefore, we reasoned that if an additive was present during concentrating that could ameliorate the aggregation, then differences in flow-rates between additives during spin-concentrating would serve as the assay (Fig. 3.6b).

**Fig. 3.7:**
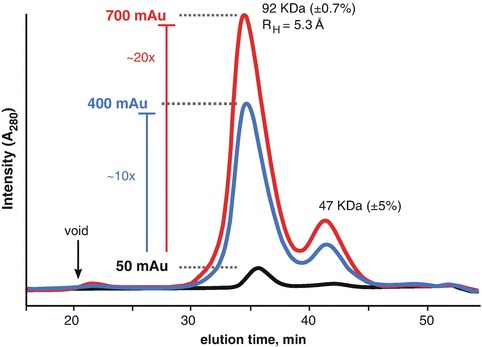
SEC-MALS analysis of WRN exonuclease in the presence of phosphate and sucrose. The A_280_ signal for the unconcentrated (*black trace*) sample increase nearly tenfold as the initial sample volume was reduced by tenfold (*blue trace*). A_280_ signal increased further (*red trace*) with a further reduction in sample volume. The peak in the void volume (*arrow*) was severely attenuated in the presence of the additives. MALS mass of the main peak (92 kDa) suggests the protein is a dimer with a monomeric mass of 47 kDa

For the WRN exonuclease domain, aliquots of protein were mixed with various additives to 600 uL. The volume was transferred to a set of spin-concentrators where the weight of each tube was pre-recorded. Flow-rates were determined by weighing each tube at intervals of 5 min during the centrifugation. We found that 50 mM phosphate produced the fastest flow rate with 2% sucrose in second place. Some additives were too slow to be considered effective. These results suggested phosphate and sucrose could be stabilizing to the protein. To validate the stabilization, the protein was concentrated in a mixture of 50 mM phosphate and 1% sucrose and subject to SEC-MALS. Ideally, if a protein is concentrated tenfold, then it can be expected there would be a corresponding increase in the A_280_ at the elution peak. In the absence of additives, concentrating the protein led to an A_280_ peak in the void volume suggesting most of the protein was forming large aggregates. However, in the presence of phosphate and sucrose (Fig. 3.7), we see that concentrating the protein by 10× increased the A_280_ nearly 10× with no increase in absorbance near the void. The stability was further demonstrated by concentrating the protein 20×. The MALS results showed the protein existed as a stable dimer and allowed for confident interpretation of the SAXS data collected from the peak.

**Fig. 3.8:**
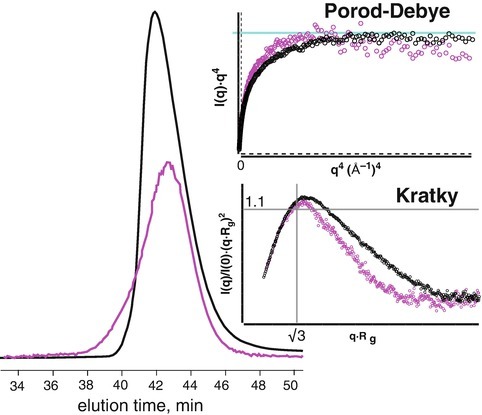
SEC analysis of a small RNA binding protein. Glycerol is a common reagent used to stabilize proteins against aggregation during freezing or concentrating. While the stability may inhibit material loss of the sample, the glycerol may not promote or inhibit non-ideal behaviour. SEC (*black trace*) and subsequent SAXS of the protein (*black circles*) in 5 % glycerol showed a protein with an asymmetric elution profile. SAXS data indicated the protein was compact with a discrete electron density contrast (plateau in the Porod-Debye plot, *cyan*). However, dimensionless Kratky plot showed a peak that was not globular. Globular proteins exhibit a peak at the Guinier-Kratky point (√3, 1.1). Purifying the protein in 100 mM phosphate and 2% sucrose (substituting glycerol), caused a notable shift in the SEC QELS peak (*magenta*). The peak shows a stable shoulder. SAXS analysis in the new condition revealed a stable Porod-Debye plateau and a shift of the SAXS peak towards the Guinier-Kratky point. The results demonstrate that the thermodynamic state of the protein can be modulated using additives

Similar results were obtained with a small, 37.5 kDa RNA binding domain. The protein was purified for crystallization and concentrated in 5% glycerol. The glycerol was necessary to keep the protein “happy”. However, SEC analysis reveal an asymmetric elution peak suggesting heterogeneity and further SAXS analysis of the SEC purified protein implied a protein with a volume of 99,687 Å^3^, far too large for a monomer and far too small for a dimer. This ambiguity suggests the protein is a mixture of monomeric and dimeric states. We found the protein required 100 mM phosphate and 2% sucrose to be stable to SEC partitioning. QELS analysis in the phosphate-sucrose buffer showed an elution peak with a distinct leading shoulder (Fig. 3.8). SAXS of the main peak determined a particle volume of 84,000 Å^3^ suggesting the heterogeneity of the sample could be influenced by additives. In both conditions, the SAXS sample displayed a distinct plateau in the Porod-Debye plot supporting the presence of a compact, well-folded particle (Rambo and Tainer 2011) but it was the discrepancy between the experimental volume and expected mass that confirmed the suspected heterogeneity. It can be expected that a buffer additive that alters the plateau region in the Porod-Debye plot will also effect the peak profile in a dimensionless Kratky plot.

We have found that additives such as pH, sucrose, sulfate, phosphate, ATP, GTP, proline, arginine or heparin-like sulfated carbohydrates to be effective additives to a wide range of proteins. Sulfate or sulfated-carbohydrates were effective in stabilizing extracellular matrix proteins whereas ATP/GTP-vanadate was important for specific motor proteins including dynamin and DNA repair proteins. If the protein of interest contains a Walker A/B motif, it may be important to lyse the cells in a high phosphate containing buffer as the phosphate ions may slow the release of nucleotide diphosphate (a simple application of Le Chatelier’s principle).

## Choosing the Right Column

**Fig. 3.9:**
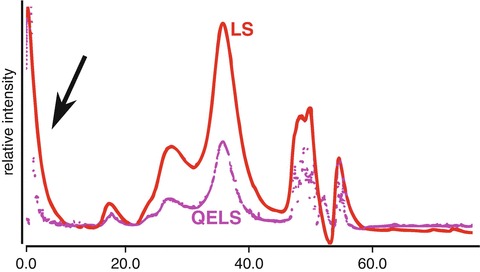
Rapid pressurization of an SEC column is damaging to the resin. Initial pressurization will degrade the resin and cause debris to elute from the column. This debris can cause considerable light scatter (LS) in the beginning of the column run (*black arrow*) and leads to an elevated baseline. We recommend starting a column at a low flow-rate and incrementing by doubling until the desired flow-rate is achieved. The operating flow-rate should be maintained in a continuous flow-mode until the experimental session ends

SEC columns have a useful separation range that is described in terms of molecular weights. As mentioned previously, there can be a linear relationship between particle mass and Stoke’s radius, but a proper description of separation range would be in terms of Stoke’s radius. The ubiquitous Superdex 75, 200 and Superose 6 columns (GE Healthcare Life Sciences) have dominated SEC of biological macromolecules. These columns contain polymeric resins derived from cross-linked agarose and are chemically inert. The Superdex 75 is recommended for proteins less than 70 kDa, whereas the Superdex 200 is recommended for proteins less than 200 kDa and the Superose 6 is for complexes that are less than 5,000 kDa. These resins are compressible and can experience pressure-induced degradation during the initial start-up of the HPLC system. The degradation leads to the loss of material from the column and if connected to a MALS instrument, there will be a large scattering signal during the beginning of the chromatographic run (Fig. 3.9). It is recommended that SEC columns are gradually brought to the operating flow-rate and that the operating flow-rate is maintained continuously during the experimental session.

**Fig. 3.10:**
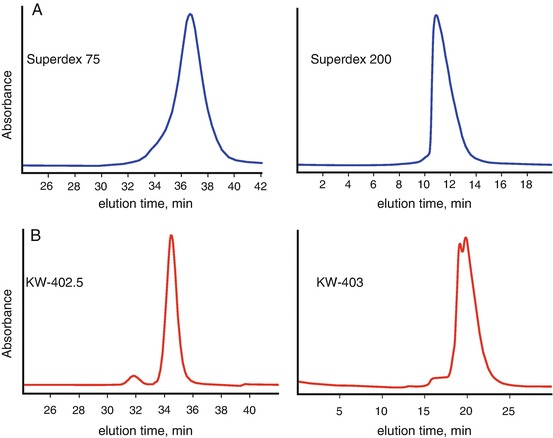
Comparison of SEC columns. (**a**) SEC analysis of xylanase (*left panel*) using Superdex 75 shows an elution peak with a leading shoulder and likewise, SEC analysis of a larger protein X (*right panel*) using Superdex 200 shows an asymmetric peak that leans towards the void volume. (**b**) SEC Analysis of the same sample on the same day using the Shodex columns shows resolution of the leading shoulder in the xylanase sample and partial resolution of protein X into two distinct peaks. Superdex columns use cross-linked agarose resins that are chemically robust but have fewer theoretical plates available for sample partitioning during SEC separation. For protein X, the peaks are not fully resolved and further analysis may require additive screening to promote a single state

Alternatively, there is another class of SEC columns growing in popularity. These are silica-based resins that use highly refined porous silica-beads. The beads can withstand greater operating pressures without sacrificing separation resolution but have a narrower operating pH range (pH <8.0). The KW-402.5, -403 and -404 columns (Shodex) offer similar separation ranges as the Superdex/Superose columns. However, the silica-based columns operate with a greater number of theoretical plates and can resolve smaller Stoke’s radii differences (Fig. 3.10). The silica-based columns contain negatively-charged silanol groups and can interact with particles differently than the Superdex/Superose columns. It can be expected at low ionic strengths, these interactions may become more influential thus changing the elution characteristics of the column. Similarly, the agarose-based resins are sugars and for carbohydrate or nucleic acid binding proteins, low-ionic strength buffer conditions (<50 mM) may promote particle-column interactions causing a noticeable tailing in the elution peak.

For SEC-SAXS, the choice of column will be determined by the mass of the particle and initial purity of the sample. If the protein elutes too close to the end of the column volume, then there is the risk of poor background subtraction as the differences in small molecules from the injection elute at the same time from the column. Likewise, if the protein elutes too close to the void, then there is the risk of contaminating the SAXS signal with scattering from large aggregates. Therefore, the choice of column should place the particle of interest away from the void volume and the end of the column volume.

## Summary

A great SAXS sample may make for a good MX sample but the reverse is not always true. Since crystallography optimizes for conditions that promote interparticle interactions, SAXS of samples prepared for crystallography must be assessed for unwanted interactions. These interactions can prevent interpretation of the solution state but can be readily attenuated using small molecule additives. In the RNA world, conditioning screening is employed early in a structural investigation as structured RNAs often require precise concentrations of divalent and monovalent metal ions (Rambo and Tainer 2010a, b; Reyes et al. 2014). Similarly, it is recommended that in the early stages of a SAXS investigation, that additive screening be explored for difficult samples as illustrated with the WRN exonuclease. The choice of buffer condition should be one that minimizes particle-column interactions while optimizing for stability.

SEC-coupled SAXS is available at most synchrotron facilities that focus on SAXS of biological samples in the solution-state (Bizien et al. 2016). These experiments may not be amenable to high-throughput SAXS but offer the most reliable method for collecting quality SAXS data. Since the measurement is under-flow, the resulting SAXS curve will be an accumulation of short exposures that may not be sufficient to capture the SAXS curve at moderately high scattering vectors (*q* > 0.2 Å^−1^). Repeated measurements of the same sample, slower flow-rates or static samples (batch) with increased exposure times would allow for data collection to higher *q*-values. If preparing samples for batch mode (PCR strips or 96-well plates), sample preparation using SEC is optimal but does not guarantee perfect background subtraction. As mentioned previously, collecting samples near the end of the column volume may lead to a buffer mismatch and purifications schemes should be employed that push the particle of interest away from the end of the column volume. More importantly, the buffer that is collected for the background measurement must be treated just as special as the sample containing the protein. Keeping the buffer at a different temperature or exposed to air while the protein sample is stored on ice will allow for different oxidation rates. These difference are noticeable in a reducing environment (DTT, TCEP, BME) and can be a major source of buffer mismatching.
